# Midkine—A novel player in cardiovascular diseases

**DOI:** 10.3389/fcvm.2022.1003104

**Published:** 2022-09-20

**Authors:** Marina Majaj, Ludwig T. Weckbach

**Affiliations:** ^1^Walter Brendel Centre for Experimental Medicine, Biomedical Centre, Institute for Cardiovascular Physiology und Pathophysiology, Ludwig-Maximilians-University Munich, Munich, Germany; ^2^Department of Neurology, Heidelberg University Hospital, Heidelberg, Germany; ^3^Medizinische Klinik und Poliklinik I, Klinikum der Universität München, Munich, Germany; ^4^Deutsches Zentrum für Herz-Kreislauf-Forschung e. V, Berlin, Germany

**Keywords:** midkine, cytokine, inflammation, cardiovascular disease, biomarkers, therapeutic targets

## Abstract

Midkine (MK) is a 13-kDa heparin-binding cytokine and growth factor with anti-apoptotic, pro-angiogenic, pro-inflammatory and anti-infective functions, that enable it to partake in a series of physiological and pathophysiological processes. In the past, research revolving around MK has concentrated on its roles in reproduction and development, tissue protection and repair as well as inflammatory and malignant processes. In the recent few years, MK's implication in a wide scope of cardiovascular diseases has been rigorously investigated. Nonetheless, there is still no broadly accepted consensus on whether MK exerts generally detrimental or favorable effects in cardiovascular diseases. The truth probably resides somewhere in-between and depends on the underlying physiological or pathophysiological condition. It is therefore crucial to thoroughly examine and appraise MK's participation in cardiovascular diseases. In this review, we introduce the MK gene and protein, its multiple receptors and signaling pathways along with its expression in the vascular system and its most substantial functions in cardiovascular biology. Further, we recapitulate the current evidence of MK's expression in cardiovascular diseases, addressing the various sources and modes of MK expression. Moreover, we summarize the most significant implications of MK in cardiovascular diseases with particular emphasis on MK's advantageous and injurious functions, highlighting its ample diagnostic and therapeutic potential. Also, we focus on conflicting roles of MK in a number of cardiovascular diseases and try to provide some clarity and guidance to MK's multifaceted roles. In summary, we aim to pave the way for MK-based diagnostics and therapies that could present promising tools in the diagnosis and treatment of cardiovascular diseases.

## Introduction

### Gene and protein

Midkine (MK) was discovered in the course of retinoic-acid-mediated differentiation of murine embryonic carcinoma cells during early stages of embryogenesis (1). Whereas, the human MK gene (*MDK*) is found on chromosome 11 at p11.2, the murine MK gene (*Mdk*) is located on chromosome 2 (2, 3). There exist 4 coding exons in *MDK* and 7 isoforms of MK messenger ribonucleic acid (mRNA) (4, 5). A retinoic acid response element and a hypoxia-responsive element as well as binding sites for the product of Wilms tumor suppressor gene WT-1 and nuclear factor kappa-B (NF-κB) are incorporated in *MDK*'s promotor region (5–8). Additionally, MK expression was elevated by tumor necrosis factor alpha (TNFα) *via* NF-κB pathway (9). Consistently, glucocorticoids, renowned for their anti-inflammatory influence, downregulated MK expression (10). Hence, MK expression is enhanced upon hypoxia and inflammation, which goes in line with its previously described roles in inflammatory processes and tumorigenesis (11–14).

**Figure 1:**
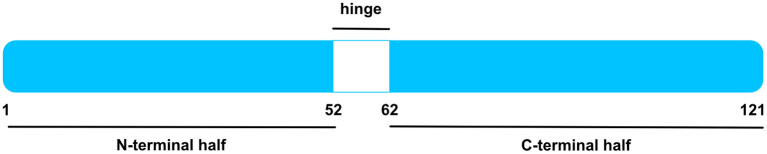
Human MK Protein. Schematic illustration of human MK protein, after signal sequence cleavage. MK is compiled by an N-terminal and a C-terminal half, signified by blue boxes. Whereas, the former entails the N-tail (aa 1–14) and N-domain (aa 15–52), the latter is composed by the C-domain (aa 62–104) and C-tail (aa 105–121). The hinge region in white (aa 53–61) connects both domains. The C-domain contains several heparin-binding sites. Figure was adapted from Weckbach et al. (14).

MK and Pleiotrophin (PTN) share ca. 50% sequence homology and compile a heparin-binding growth factor family (4, 15). Both proteins are conserved across a wide range of vertebrates (4, 15). For instance, murine and human MK have 87% amino acid sequence homology (Figure 1) (16). Moreover, zebrafish express two MK molecules known as Midkine-a and Midkine-b (17) and *Drosophila melanogaster* express proteins, miple 1 and miple 2, that bear resemblance to C-domains of MK and PTN, respectively (18).

As specified in the Uniprot Database [UniProtKB—P21741 (MK_HUMAN)], the 15.5 kDa human MK protein [amino acids (aa) 1–143] is a secreted protein and contains a signal peptide (aa 1–20). Upon signal sequence cleavage, the remaining main protein chain has 121 amino acids and holds a molecular mass of 13 kDa (4, 16). A number of MK isoforms were identified such as VA-MK, an extended form of MK with additional N-terminally located valine and alanine (4, 19) and several truncated MK isoforms (13, 20, 21). The MK protein entails two domains, namely an N-terminally positioned N-domain (aa 15–52) and a C-terminally positioned C-domain (aa 62–104) (4, 22). Each domain comprises 3 anti-parallel β-strands held together by disulfide bridges (4, 23). Both domains are bound by a hinge region (aa 53–61) and are flanked by an N-tail (aa 1–14) and a C-tail (aa 105–121) at their respective ends (4). MK is enriched with basic amino acids and cysteines, rendering it positively charged with a heparin-binding capability (4, 23). Two heparin-binding sites were identified in the C-domain (4, 23, 24). Apart from its heparin-binding quality, the C-domain was found to be implicated in most of MK's functions including neurite promoting activity (4, 24, 25) and enhancement of plasminogen activator activity (4, 25, 26). Moreover, the MK receptor protein tyrosine phosphatase receptor type zeta 1 (PTPRZ1) binds the C-terminal half (27). Contrariwise, the N-domain appears to confer stability, for the C-terminal half was more prone to chymotrypsin digestion when compared to intact MK (4, 28). Furthermore, the N-domain was crucially involved in MK-mediated neutrophil trafficking and cardiac inflammation *via* the MK receptor low-density lipoprotein receptor-related protein 1 (LRP) 1 (29).

Interestingly, some investigations unraveled the expression of MK dimers in an array of tissues (30, 31). MK dimerization was mediated by transglutaminase crosslinking (32, 33). In the presence of heparin, MK dimerization was augmented (32). Of note, the dimer form of MK was designated as the biologically active form. This was shown for MK dimer's role in promoting fibrinolysis through activating plasminogen activator (32), as well as in supporting neurite outgrowth (30).

### Receptors and signaling

MK utilizes numerous receptors to exert its functions in a wide scope of physiological and pathophysiological processes (Table 1). Remarkably, it was demonstrated that a functional MK receptor complex can be assembled by multiple MK receptors and that MK fostered receptor complex formation (4, 42, 56). This observation is in consonance with the aforementioned critical role of MK dimers for its functions, as a dimer may encourage the formation of a receptor complex (4). Furthermore, certain MK receptors seem to share common signaling pathways (57).

**Table 1:** Summary of MK receptors and their roles in MK functions.

**Receptor**	**MK function mediated by receptor**	**References**
Protein tyrosine phosphatase	↑ Embryonic neuron migration	(27)
Z1 (PTPRZ1)	↑ Osteoblast-like cell line UMR-106 migration	(34)
	↑ Embryonic neuron survival	(35)
	↑ B cell survival	(36)
	↓ Osteoblast proliferation	(37)
Receptor low-density	↑ Chondrocyte proliferation	(38)
lipoprotein receptor-related	↑ Embryonic neuron survival	(39)
protein 1 (LRP1)	↓ Hypoxic injury in embryonic stem cells	(40)
	↑ Neutrophil recruitment	(41)
	↑ Neutrophil recruitment, neutrophil extracellular traps (NET) formation, and cardiac inflammation in murine myocarditis model	(29)
α_4_β_1_	↑ Osteoblast-like cell line UMR-106 migration	(42)
α_6_β_1_	↑ Neurite outgrowth of embryonic neurons	(42)
	↑ Human head and neck carcinoma cell migration and invasiveness	(43)
Notch2	↑ Immortalized HaCaT keratinocytes epithelial-mesenchymal transition	(44)
	↑ Pancreatic ductal adenocarcinoma cell epithelial-mesenchymal transition and chemoresistance	(45)
	↑ Neuroblastoma development	(46)
	↑ Human lung epithelial cell epithelial-mesenchymal transition	(47)
	↑ Vascular endothelial injury	(48)
Anaplastic lymphoma kinase (ALK)	↑ Adrenal gland tumor cell line SW-13 growth	(49)
	↑ Immature sympathetic neurons proliferation	(50)
	↑ Glioblastoma cell cannabinoid resistance	(51, 52)
Syndecans and glypican-2	↑ Neuronal development migration and growth	(53, 54)
Neuroglycan C	↑ CG-4 oligodendroglial precursor-like cells elongation	(55)

PTPRZ1 is one of the most established MK receptors. Downstream of PTPRZ1, tyrosine phosphorylation of numerous cytoplasmic signaling molecules including β-adducin (58), β-catenin (37), Fyn (59), Syk (36) and Akt (34, 36) was observed. Moreover, the kinases phosphatidylinositol 3 kinase (PI3K), mitogen-activated protein kinase (MAPK), and protein kinase C (PKC) similarly participated in signaling downstream of PTPRZ1 (34). Members of low-density lipoprotein receptor family, LRP1 (39), megalin (39) and ectodomains of LRP-6 and apo-E receptor-2 (35) also served as MK receptors. Among them, LRP1 bound MK with the highest affinity (39). LRP1 facilitated MK endocytosis and consequent nuclear targeting through the shuttle protein nucleolin (38, 60). Another study described signal transmission *via* Akt and hypoxia-inducible factor 1-alpha in the MK-LRP1 axis (40). Furthermore, we could show that LRP1 was involved in MK-mediated neutrophil recruitment, specifically *via* promoting conformational changes of β_2_-integrins (41). In a follow up study, we unveiled the role of MK-LRP1 axis in neutrophil extracellular trap (NET) formation (29). In addition, the integrins α_4_β_1_ and α_6_β_1_ operated as MK receptors (42). It is noteworthy that LRP6 ectodomain, PTPRZ1, α_4_β_1_ and α_6_β_1_ assembled a MK receptor complex (42). Also, MK mediated tyrosine phosphorylation of paxillin, which is implicated in integrin downstream signaling (42). Another report provided evidence of MK binding to tetraspanin and α_6_β_1_ and consequent tyrosine phosphorylation of focal adhesion kinase and activation of paxillin and signal transducer and activator of transcription proteins (STAT) 1α pathways (43). Moreover, the transmembrane protein Notch2 was classified as a MK receptor. Further analysis showed that MK stimulated Notch2-Jak2-STAT3 signaling (44). Another study described MK-mediated cleavage of Notch2 cytoplasmic domain and elevated expression of hairy and enhancer of split-1 and NF-κB (45). Also, downstream of MK-Notch2, an upregulation of ACE expression has been noted (47, 48). Another crucial MK receptor is the transmembrane tyrosine kinase anaplastic lymphoma kinase (ALK) (49). It has been proposed that ALK, LRP and integrins can compile a MK receptor complex (56) and activation by MK led to phosphorylation of insulin receptor substrate-1 by ALK, as well as activation of MAPK, PI3K and NF-κB (49, 61). Signal transduction through MAPK and PI3K thus represents a common signaling pathway since it was demonstrated for MK-PTPRZ1 axis, as mentioned above. Finally, MK can also bind glycosaminoglycans such as the heparan sulfate proteoglycans syndecans (53) and glypican-2 (54), the chondroitin sulfate neuroglycan C (55) and heparan sulfate-trisulfated units and chondroitin sulfate E (62).

### MK expression in cells of the vascular system

While MK expression during murine embryogenesis was particularly upregulated in mid-gestation stage, it was confined to the kidney in adult mice (63). In humans, MK expression was analyzed in various processes. MK was highly expressed in malignant and inflammatory settings (12–14, 64). In the sera of healthy volunteers, MK expression was also detected (65). Compellingly, MK is classified as a heparin-releasable protein (19). Intravenous heparin administration significantly elevated MK serum levels with a peak exhibited at 10 to 30 min (19, 66). Post-heparin MK level increase was even dose-dependent (66). In this context, heparin-releasable MK did not stem from blood cells or the kidney (19). It is assumed that endothelial cells (ECs) may represent a source of post-heparin MK (19). This is supported by the observation that heparin-releasable proteins, equipped with their heparin-binding sites, could reside in the luminal surface of the endothelium (19). Heparin could therefore compete for these sites on the EC surface and cause the liberation of heparin-releasable proteins (57). Moreover, ECs produced MK under resting conditions (19). Consistently, under hypoxic conditions, MK expression was not only augmented, but MK was also secreted by ECs (67). Furthermore, neutrophils and monocytes displayed elevated MK expression upon hypoxia, but did not release MK (67). In addition, MK expression in other cells of the vascular system such as macrophages (68) and lymphocytes (36, 69, 70) was illustrated. On the contrary, a different study could not verify MK expression in lymphocytes and monocytes (71). Given the copious diagnostic and therapeutic aptitude of MK, it is imperative to divulge the source and regulation of its expression in the vascular system.

### Midkine in cardiovascular biology

#### Angiogenesis and arteriogenesis

Amid mammalian embryo development, vasculogenesis occurs when angioblasts, that differentiate into ECs, create a vascular labyrinth (72, 73). Angiogenesis defines the consequent sprouting that assembles a vascular network which could develop into arteries and veins (72, 73). The recruitment of vascular smooth muscle cells and pericytes which cover ECs designates the process of arteriogenesis, critical for perfusion control and stability (72, 73).

MK played a vital role in angiogenesis. MK was over expressed in MCF-7 breast carcinoma cells and stimulated tumor growth, *in vivo* (74). Here, MK prompted EC proliferation and instigated an increase in vascular density (74). The robust angiogenic effect of MK was further confirmed in a rabbit corneal assay (74). Moreover, human umbilical vein endothelial cell (HUVEC) proliferation, *in vitro*, was amplified by conditioned media of the MK-overexpressing human bladder cancer UM-UC-3/MK cell line (75). *In vivo*, a growth advantage associated with increased micro-vessel density was exhibited following subcutaneous and orthotopical administration of UM-UC-3/MK cells in nude mice (75). Furthermore, antisense oligonucleotide targeting MK inhibited HUVEC growth, *in vitro*, and hindered angiogenesis in chick chorioallantoic membrane (CAM) assay and in human hepatocellular carcinoma xenograft, *in situ* (76). MK's contribution in hepatocellular carcinoma angiogenesis was further examined (77). In this perspective, MK was able to bind the glycoprotein Progranulin and through this interaction stimulated HUVEC proliferation, migration and tubulogenesis (77). As explored by the chick CAM assay, an increase in the angiogenic response upon incubation with MK and Progranulin was shown (77). In addition, another report revealed that combinational treatment with small interfering RNA (siRNA) against MK and paclitaxel effectively suppressed human prostate cancer PC-3 cell line angiogenesis (78). In line with these studies, using a chick CAM assay, we demonstrated that exogenous MK boosted neovascularization, in a concentration dependent manner, when compared to the negative control (67). Similarly, in a murine hind limb ischemia model, angiogenesis was extremely compromised in MK-deficient mice, relative to wildtype mice (67). Further, a new study unveiled elevated MK expression in mice lacking protease nexin-1, known for its anti-angiogenic activity, in whom femoral artery ligation was induced (79). In these mice, rapid artery perfusion, elevated capillary density, increased leukocyte recruitment and interleukin-6 and monocyte chemoattractant protein-expression was observed (79). Here, MK upregulation had a positive effect on neoangiogenesis in response to ischemia.

Congruent to MK's involvement in angiogenesis, MK is additionally involved in arteriogenesis (80). MK's promotion of EC proliferation of growing collateral arteries was unraveled in a murine hind limb model applied in MK-deficient and wildtype mice (80). Leukocyte-derived MK was accountable for collateral artery growth and arteriogenesis (80). Whereas, leukocyte-domiciled MK increased vascular endothelial growth factor A plasma levels, important for endothelial nitric oxide synthase 1 and 3 upregulation, non-leukocyte domiciled MK additionally improved vasodilation (80).

#### Host defense

Relying on its anti-bacterial and anti-viral properties, MK participated in host defense. MK bears similarities with anti-bacterial proteins, like β-defensins and the anti-bacterial chemokines (81). Common structural features such as cationic quality and the MK domains built by three anti-parallel β-sheets, heparin-binding capacity and the ability of oligomerization particularly enabled MK to partake in anti-bacterial host defense (81). In this regard, MK displayed potent bactericidal activity against gram-positive bacteria such as *Staphylococcus aureus, Streptococcus pneumoniae* and *Streptococcus pyogenes* and gram-negative bacteria such as *Pseudomonas aeruginosa* and *Escherichia coli* (82–85). This bactericidal property was attributed to MK-induced bacterial membrane disruption (82). On the other hand, MK exerted anti-viral functions. The MK-mediated inhibition of human immunodeficiency virus cell infection was demonstrated by one study (86). In a dose dependent manner, MK obstructed T-lymphocyte- and macrophage-tropic human immunodeficiency virus-1 cell surface binding and interfered with its internalization (86).

#### Recruitment of leukocytes

The MK function in facilitating inflammation has been thoroughly investigated in a wide scope of inflammatory processes (12, 14, 64). Briefly, MK was vastly expressed in inflamed tissue and elevated MK levels were measured in patients with various inflammatory diseases (12, 14, 64). Furthermore, MK-deficient mice presented with milder disease manifestation, when compared to wildtype mice, in multiple inflammatory disease animal models (12, 14, 64). Moreover, while MK administration aggravated disease manifestation, targeting MK alleviated disease presentation (12, 14, 64). Several investigations collectively determined that MK primarily drived inflammation *via* leukocyte recruitment. There is a solid line of evidence for MK-stimulated recruitment of neutrophils and macrophages (29, 41, 87–96). Some reports also shed light on MK's interference in lymphocyte-associated pathologies (69, 97–99). Early evidence of MK-stimulated neutrophil recruitment revealed that MK functioned in a chemotactic manner, stimulating neutrophil migration across a concentration gradient, and a haptotactic manner, where substrate bound MK was the active form (87). In our previous work, we were able to confirm that immobilized MK supported neutrophil adhesion *via* promoting the high affinity conformation of β-integrins on neutrophils *via* its receptor LRP1 (41). Other mechanisms of MK-induced leukocyte recruitment involve the induction of macrophage inflammatory protein-2 and macrophage chemotactic protein-1 chemokines, instigating neutrophil and macrophage recruitment, respectively (91, 94).

## Discussion: Midkine in cardiovascular diseases

**Figure 2:**
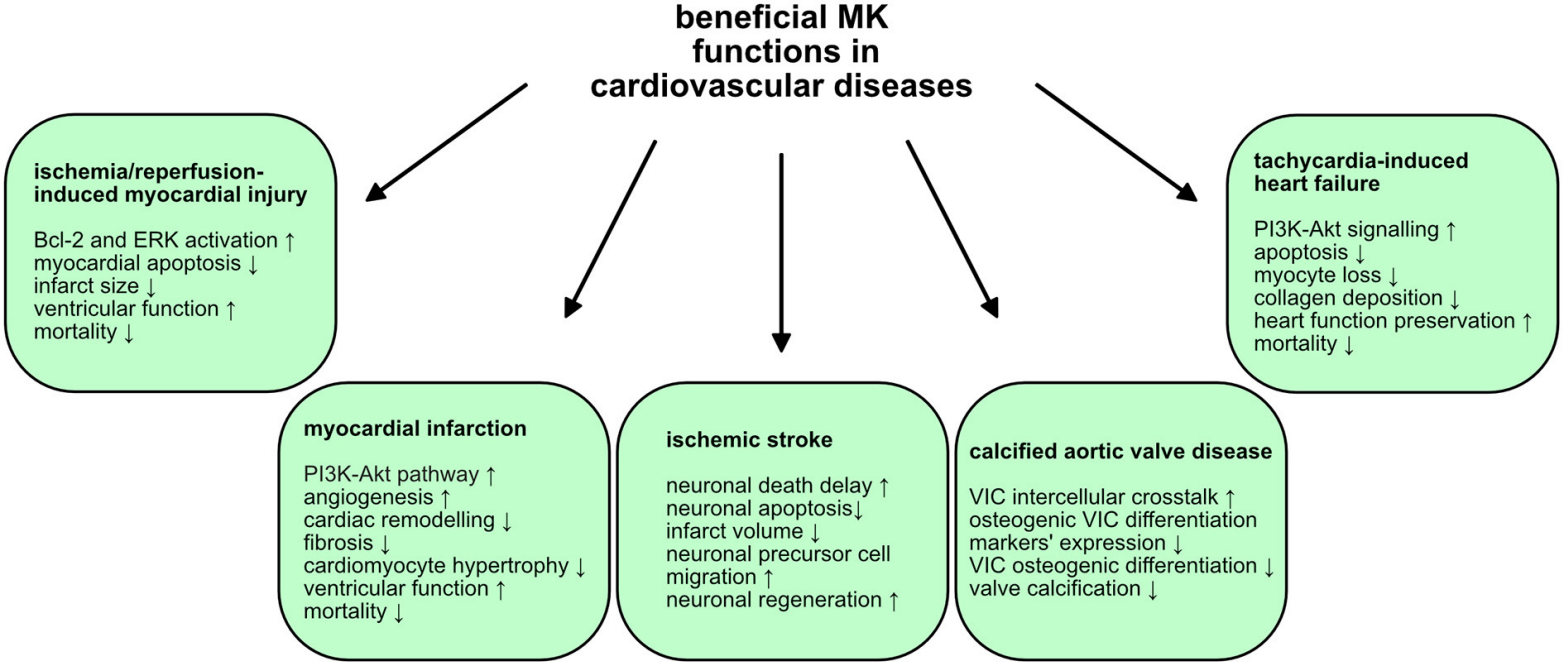
Beneficial MK functions in cardiovascular diseases. MK exercises advantageous and protective functions in some cardiovascular diseases. The mechanisms and signaling pathways utilized by MK are illustrated.

**Figure 3:**
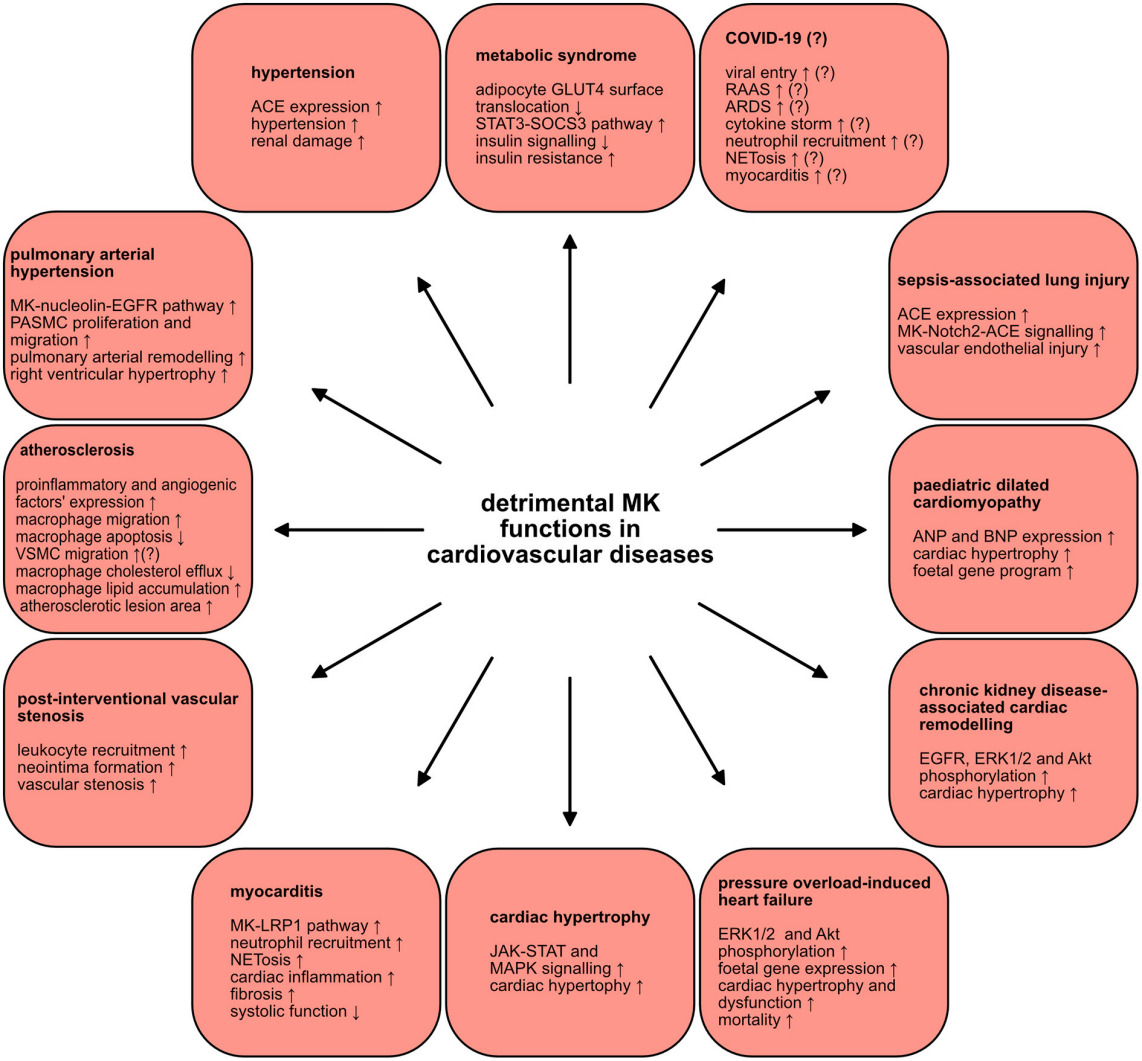
Detrimental MK functions in cardiovascular diseases. MK plays injurious roles in a wide scope of cardiovascular diseases. The various mechanisms and signaling pathways through which MK negatively contributes to the pathogenesis of several cardiovascular diseases are depicted.

MK is involved in several cardiovascular diseases and elevated MK expression was noted by numerous studies of cardiovascular diseases, illustrated in (Table 2). MK exercises both beneficial (Figure 2) and detrimental (Figure 3) functions in the pathologies of cardiovascular diseases. This section provides a summary of the expression and involvement of MK in cardiovascular diseases.

**Table 2:** MK expression in cardiovascular diseases.

**Disease**	**Elevated MK expression**	**References**
Metabolic syndrome	Adipocytes (3T3-L1 preadipocytes), adipose tissue of obese ob/ob mice, sera of overweight and obese human subjects	(100)
Hypertension	Sera of essential hypertension patients	(101)
	Plasma, lungs and kidneys of mice with 5/6 nephrectomy-induced hypertension	(102)
Pulmonary arterial hypertension (PAH)	Sera of PAH patients, lungs and sera of mice with hypoxia-induced PAH	(103)
Peripheral artery disease (PAD)	Smooth muscle cells, inflammatory and EC of thickened intima of fatty-streaks in lesions from PAD patients, extracellular matrix and intimal cells in advanced lesions from PAD patients	(104)
	Sera of severe PAD patients	(105)
	Plasma of diabetic patients with PAD	(106)
Coronary artery disease (CAD)	Plasma of CAD patients with significant stenosis	(107)
	Sera of heparin-treated CAD and acute coronary syndrome patients	(66)
Post-interventional vascular stenosis	Neointima and macrophages in hypercholesterolemic rabbits with bare metal stent implantation in atheromatous lesions	(68)
	Neointima of carotid artery in rats with intraluminal balloon injury	(88)
Ischemic myocardial injury	Periinfarct area of mice with coronary artery ischemia/reperfusion (I/R)-induced injury	(108)
	Periinfarct area in swine with left anterior descending coronary artery I/R- induced injury	(109)
	Left ventricular tissue in mice with left coronary artery ligation-induced myocardial infarction (MI)	(110)
	Infarcted myocardium in rats with MI	(111)
	Sera of patients with post-MI cardiac remodeling	(112)
Ischemic stroke	Reactive astrocytes in rats with transient cerebral ischemia	(113)
	Peri-infarct penumbra of cerebral cortex in rats with electroacupuncture intervention following middle cerebral artery occlusion/reperfusion	(114)
	Brain tissue in rats with preconditioning exercise prior to ischemic stroke	(115)
Calcified aortic valve disease (CAVD)	Matrix valvular interstitial cells from human aortic valves	(116)
Myocardial inflammation	Cardiac tissue in mice with experimental autoimmune myocarditis (EAM)	(29)
Heart failure	Sera of heart failure patients, sera of heart failure patients with cardiac events	(117)
	Sera of heart transplant recipients	(118)
	Urine of patients post-cardiac surgery with severe fluid overload	(119)
	Lung and kidney in mice with transverse aortic constriction (TAC)-induced heart failure	(120)
	Sera and cardiac tissue of dilated cardiomyopathy (DMC) pediatric patients	(121)
	Epicardium of zebrafish heart with cryoinjury	(122)
Sepsis	Sera of patients with sepsis and septic shock, sera of septic patients with cardiovascular insufficiency and mechanical ventilation, gram-positive bacterial infection-associated sepsis	(123)
	Plasma of septic patients with moderate/severe acute respiratory distress syndrome (ARDS) and acute kidney injury, plasma of non-survivor group of septic patients	(124)
	Plasma of septic patients, plasma and lung in mice with cecal ligation and puncture-induced sepsis	(48)
Coronavirus disease 19 (COVID-19)	Plasma of COVID-19 patients	(125)
	Sera of pregnant women with COVID-19	(126)
	Sera of pregnant women with COVID-19, sera of pregnant women with moderate and severe COVID-19	(127)

### Metabolic syndrome

The metabolic syndrome is characterized by a constellation of several metabolic disorders. These comprise abdominal obesity, insulin resistance, dyslipidaemia (elevated levels of triglycerides and low levels of high-density lipoprotein), hypertension as well as a pro-inflammatory, pro-thrombotic milieu (128). These metabolic abnormalities are a well-established risk factors for type 2 diabetes and cardiovascular diseases (128). Since there is an undeniable inflammatory component in obesity (129), a connection between MK and obesity was proposed. Indeed, MK was expressed in adipocytes and its expression was modulated by inflammatory mediators (100). Whereas, MK expression was induced *via* TNFα, rosiglitazone, known to inhibit inflammation in adipocytes, diminished MK expression (100). Analysis of adipose tissue of obese ob/ob mice, a murine model of severe genetic obesity and insulin resistance, uncovered profound MK expression relative to control mice (100). Congruently, serum MK levels in overweight and obese human subjects were significantly elevated as opposed to healthy controls (100). Interestingly, a positive correlation between body mass index (BMI) and MK was demonstrated (100). In depth analysis of MK's functional relevance in obesity disclosed impairment of insulin signaling in adipocytes upon MK exposure (100). Remarkably, MK hindered the insulin-stimulated translocation of the glucose transporter GLUT4 to the surface of adipocytes and activated the STAT3-suppressor of cytokine signaling 3 pathway, implicated in adipocyte insulin resistance (100). Hence, MK might pose a possible link between obesity and insulin resistance. On another note, some authors even hypothesized about the existence of a putative relationship between MK and the oral anti-diabetic medication metformin (130). Here, it was suggested that metformin could function as a potent MK inhibitor, since both have contrasting and divergent influences on numerous signaling pathways and biological processes (130). If proven true, this could further substantiate the detrimental role of MK in the development of insulin resistance. Taken together, MK might contribute to the pathogenesis of the metabolic syndrome.

### Hypertension

MK was also linked to hypertension. Serum MK expression was significantly elevated in patients with essential hypertension, in contrast to healthy subjects (101). MK levels exhibited a positive correlation with diastolic and systolic blood pressures (101). The impact of MK in the hypertension pathophysiology *via* regulation of the renin angiotensin aldosterone system (RAAS), notorious regulator of blood pressure, was clarified. In the aorta of MK-deficient mice, renin and angiotensinogen expression was augmented, while angiotensin converting enzyme (ACE) expression was suppressed (131). Strikingly, MK-deficient mice did not develop hypertension and displayed systolic and mean blood pressure analogous to wildtype mice (102). In a murine model that RAAS-dependently provokes hypertension as a result of 5/6 nephrectomy, ACE expression was significantly increased in the lungs of wildtype mice and a rise in MK expression in their plasma, lungs and kidneys was detected (102). On the contrary, MK-deficient mice were protected against hypertension and were shielded from the resultant renal damage (102). In alignment with this, supplementary MK application in MK-deficient mice, who underwent 5/6 nephrectomy, restored hypertension, and prompted pulmonary ACE expression (102). Consistently, treatment of primary cultured human lung microvascular ECs with MK enhanced ACE expression, *in vitro* (102). In addition, NADPH oxidases 1, −2 and −4 were upregulated in wildtype mice, in contrast to MK-deficient mice, indicating that MK fuelled oxidative stress in this model (102). Conversely, treatment with an anti-oxidative agent decreased pulmonary MK expression, plasma angiotensin II levels and blood pressure (102). This suggests that oxidative stress could also induce MK expression, as was reported by other studies (91, 93). These results set forth a probable vicious cycle of the interplay between MK, ACE, angiotensin II and oxidative stress that ultimately exacerbates hypertension caused by 5/6 nephrectomy (102). In conclusion, MK might be an attractive target for establishing anti-hypertensive therapies.

### Pulmonary arterial hypertension

The chronic and progressive cardiopulmonary disease, pulmonary arterial hypertension (PAH), is characterized by small pulmonary artery proliferation and fibrosis which ultimately triggers a rise in pulmonary vascular resistance (132). Recently, a report described a novel MK role in PAH (103). In the sera of patients with PAH, MK concentrations were drastically higher relative to control patients (103). MK was also upregulated in lungs and sera of mice suffering from hypoxia-provoked PAH (103). When hypoxia-induced PAH was conducted in MK-deficient mice, right ventricular hypertrophy and pulmonary arterial remodeling were alleviated (103). Under hypoxic conditions, pulmonary arterial smooth muscle cells migration and proliferation were enhanced by MK *via* the nucleolin-epidermal growth factor receptor (EGFR) pathway (103). Here, hypoxia stimulated cell surface translocation of nucleolin as well as MK-mediated EGFR signaling (103). Consistently, suppression of nucleolin protected against PAH development *via* hindering the MK-nucleolin-EGFR axis (103). Henceforth, MK contributed to PAH pathogenesis and could be a new candidate target in PAH treatment.

### Atherosclerotic cardiovascular disease

The development of atherosclerotic plaques, fibrofatty lesions in the intimal layer of arteries, is referred to as atherosclerosis (133). Advanced plaques grow toward the artery lumen and can obstruct blood flow causing tissue ischemia (133). The non-obstructive plaques can rupture and subsequently cause vessel impediment leading to acute tissue ischemia (133). Clinically, atherosclerotic cardiovascular disease can present as coronary artery disease (CAD), acute coronary syndrome including myocardial infarction (MI), peripheral artery disease (PAD) and ischemic stroke (133).

MK expression in atherosclerosis was explored by some studies. Assessment of atherosclerotic lesions originating from PAD patients uncovered MK expression in the thickened intima of fatty-streaks, particularly in smooth muscle cells, inflammatory and ECs (104). MK was also identified in advanced atherosclerotic lesions, markedly in the extracellular matrix and in intimal cells (104). Congruently, severe PAD patients had substantial seral MK levels, as opposed to significantly lower levels in healthy volunteers (105). Surprisingly, these did not display a correlation with typical risk factors such as age, sex, obesity, diabetes mellitus, hypertension, increased cholesterol levels or smoking (105). In contrast, although not directly studied in patients with atherosclerotic cardiovascular disease, another report laid evidence for the association of raised MK levels with risk factors of atherosclerosis such as hypertension, high LDL and total cholesterol (101). The measurement of MK levels was also a part of a clinical-biomarker model for the prediction of PAD in diabetic patients (106). Moreover, in a clinical and biomarker score designed to predict the presence of significant CAD, MK was integrated as a biomarker (107). On another note, a new investigation of MK levels in patients with CAD and acute coronary syndrome, found that neither myocardial ischemia nor necrosis altered MK levels (66). Heparin administration, on the other side, robustly elevated MK levels in these patients (66). This study indicated that MK is probably not a reliable myocardial ischemia or necrosis biomarker (66). In conclusion, MK appears to be upregulated in atherosclerotic cardiovascular diseases, but further investigations are required to establish accurate and specific MK-based diagnostics.

There is increasing evidence that corroborates MK's deleterious role in atherosclerosis pathogenesis. One study utilized the prevalent murine atherosclerosis model of ApoE-deficient mice and could herewith show that systemic MK administration augmented atherosclerotic lesion area, relative to saline-treated ApoE-deficient mice (90). Likewise, mRNA expression of pro-inflammatory factors like interleukin 1 α and 1 β, interferon ^γ^ and C-C motif chemokine ligand 2, and angiogenic factors like hepatocyte growth factor and basic fibroblast growth factor in aorta of MK-treated ApoE-deficient mice were increased (90). Under these circumstances, MK promoted macrophage migration and triggered a significant increase in macrophage percentage in aortic root lesions (90). Furthermore, MK inhibited apoptosis in macrophages (90). Although without statistical significance, MK probably also contributed to the migration of vascular smooth muscle cells in atherosclerosis (90). Moreover, a recent investigation demonstrated that MK encouraged lipid accumulation in macrophages (134), critical for foam cell generation which are indispensable for atherosclerotic plaque formation (133). In this regard, MK induced the downregulation of adenosine triphosphate-binding membrane cassette transport protein A1, resulting in decreased cholesterol efflux (134). Also, adenosine monophosphate activated protein-mammalian target of rapamycin signaling was found to be involved in MK-mediated cholesterol metabolism regulation in macrophages (134). Thus, MK drove atherosclerotic plaque formation through its pro-inflammatory, pro-angiogenic and anti-apoptotic effects combined with its inhibition of macrophage cholesterol efflux. From this perspective, anti-MK therapies could attenuate atherosclerotic plaque formation.

Vascular intervention represents a widespread and effective therapy principal in atherosclerotic cardiovascular disease. Adding to its harmful effect in atherosclerosis pathophysiology, MK stimulated vascular stenosis and neointima formation following vascular intervention. In hypercholesterolemic rabbits, in whom a bare metal stent in atheromatous lesions was implanted, neointimal MK expression was induced and macrophages were identified as the MK source in the neointima (68). Consistently, a different study revealed elevated MK expression upon neointima formation succeeding rat carotid artery intraluminal balloon injury (88). Employing a murine restenosis model, neointima formation was repressed in MK-deficient mice, whilst MK administration rescued it in these mice (88). This effect was attributed to MK-mediated leukocyte recruitment in this model (88). In line with these results, another investigation proved that antisense oligodeoxyribonucleotide targeting of MK can prevent restenosis after rabbit carotid artery balloon injury (135). In addition, targeting MK in rabbit jugular vein-to-carotid artery interposition vein grafts with siRNA could even be a promising therapy for vein graft failure (89). Here, the intima thickness and intima-media ratio after grafting were diminished and accompanied by a significant decline in inflammatory cell recruitment (89). Hence, MK supported neointima formation following vascular intervention *via* leukocyte recruitment and could be a candidate target for hindering vascular restenosis.

In stern contrast to the aforementioned, undesirable MK functions in atherosclerotic cardiovascular diseases, MK does have some protective and reparative roles. MK expression was amplified in periinfarct area induced *via* ischemia/reperfusion (I/R) of coronary arteries in wildtype mice (108). Astonishingly, increased mortality, greater infarct size and myocardial apoptosis as well as reduced left ventricular fractional shortening were perceived in MK-deficient mice (108). Complementarily, exogenous MK application to the left ventricle of MK-deficient mice diminished infarct size (108). MK exerted a cardioprotective effect in this context *via* inhibiting apoptosis through Bcl-2 and extracellular signal-regulated kinase (ERK) activation (108). Additionally, an analogous study examined MK's cardioprotective role in a swine I/R model of left anterior descending coronary artery (109). At reperfusion initiation, intra-coronary MK injection shrank infarct size and alleviated ventricular function (109). Further dissection unraveled MK deposition in the periinfarct area along with a significant drop in number of apoptotic cells and, oddly, of leukocytes (109). The decline in inflammation upon MK injection could be accredited to its anti-apoptotic action, which may ameliorate secondary necrosis and concomitant inflammation (109). In a study of MK in MI, progressive elevation of MK expression in left ventricular tissue of wildtype mice, who developed MI by ligation of the left coronary artery, was disclosed (110). MK-deficient mice presented higher mortality in this model (110). Upon MK application, mortality rates were improved, left ventricular dysfunction was amended and fibrosis and cardiac remodeling were reduced in both mice strains (110). These positive MK effects were associated with its proangiogenic influence, facilitated *via* the PI3K-Akt pathway (110). In line with these observations, significant MK upregulation in the infarcted myocardium was reported in a different study of MK's role in post-MI cardiac remodeling in rats (111). In a dose-dependent manner, MK treatment, 2 weeks post-MI, profoundly weakened cardiac remodeling, mediated angiogenesis in the infarction area, augmented the area of viable muscle and lessened cardiomyocyte hypertrophy (111). On the other hand, a recent exploratory analysis of biomarkers associated with cardiac remodeling post-MI delivered evidence of a likely negative MK impact in this context (112). In patients with post-MI cardiac remodeling, MK levels were increased relative to those who didn't progress to cardiac remodeling and healthy subjects (112). Although moderate, a positive association between MK levels and left ventricular end-diastolic volume index and a negative correlation with left ventricular ejection fraction were described (112). The authors hence proposed that MK could be a useful biomarker for predicting the risk of ischemic heart failure (112). Nevertheless, it is still ambiguous whether MK elevation contributes to cardioprotection or is in itself the perpetrator of the pathology. In conclusion, MK seems to exert cardioprotective effects against myocardial injury after I/R and MI and might have a promising therapeutic potential in these settings.

### Stroke

MK exercised neuroprotective roles in ischemic stroke. MK expression was upregulated in reactive astrocytes upon transient cerebral ischemia in rats (113). Moreover, MK administration in the brain ventricle instantly prior to bilateral common carotid artery occlusion in Mongolian gerbils, delayed neuronal death 7 days following the ischemic insult (136). Furthermore, in a rat model of ischemic stroke, cerebral infarction was relieved by intraventricular injection of MK prior to ligation of middle cerebral artery ligation (137). Post-ischemic MK gene transfer also decreased infarct volume, provoked by photothrombotic occlusion-induced focal brain ischemia in spontaneously hypertensive rats (138). In this model, MK had an anti-apoptotic influence on neurons (138). In addition, applying the same model, a second report showed that post-ischemic MK gene transfer not only decreased infarct volume, but also elevated neuronal precursor cells migration toward the infarction area (139). Therefore, MK may perhaps support neuronal regeneration following ischemic brain injury (139). In a study of electroacupuncture intervention conducted in rat middle cerebral artery occlusion/reperfusion model, a reduction of infarct size, neurological deficits, and number of apoptotic cells, were observed (114). Here, MK expression was augmented in the peri-infarct penumbra of cerebral cortex and it seems that electroacupuncture could induce MK expression and hereby facilitate neuroprotection (114). Interestingly, while analyzing the neuroprotective role of preconditioning exercise in ischemic stroke in rats, enhanced MK levels were measured in comparison to the none-exercise group, corroborating MK's advantageous impact in ischemic stroke (115). In sum, MK may promote neuroprotection, which could pave the way for MK-based therapeutics in ischemic stroke.

### Calcified aortic valve disease

Calcified aortic valve disease (CAVD) is a widespread valvular heart disorder with a heterogeneous pathology (140). CAVD designates progressive aortic valve narrowing *via* active remodeling and valve mineralization, causing a decrease in valvular area and blood flow decline (140). Recently, a novel role for MK in CAVD was demonstrated. A study of CAVD disclosed that aortic matrix valvular interstitial cells (VICs) significantly expressed MK, which in a paracrine manner acted on activated and complement-activated VICs to prohibit calcification (116). Congruently, the addition of MK to the osteogenic media of VICs, isolated from aortic valve tissue from non-CAVD patients, hindered calcification (116). The expression of RUNX2 and ALP, markers of osteogenic differentiation in VICs, was diminished upon MK treatment (116). Hence, MK partakes in the inhibition of VIC osteogenic differentiation and could hereby protect against CAVD development *via* mediating intercellular crosstalk (116).

### Myocardial inflammation

The pathophysiology of the inflammatory cardiac disorder, myocarditis, is extremely heterogenous encompassing viral, bacterial, protozoal, and fungal infections, toxic substances, drugs and systemic immune diseases (141). However, myocarditis is primarily caused by viruses such as coxsackie A and B viruses, parvovirus B19, Epstein-Barr virus, and recently severe acute respiratory syndrome coronavirus 2 (SARS-CoV-2) among many others (141). Acute myocarditis can transition into an auto-immune driven disease which maintains inflammation beyond virus eradication (141). In our recent work, we delineated the influence of the innate immune system in myocarditis characterized by MK-driven neutrophil recruitment and NETosis (29). In endomyocardial biopsies stemming from myocarditis patients, NETs were detected (29). In the murine experimental autoimmune myocarditis (EAM) model, NETs were also discovered in the inflamed myocardial tissue (29). When NETosis was suppressed, cardiac inflammation, embodied by leukocyte infiltration and NET formation, was momentously weakened (29). In cardiac tissue of EAM mice, MK mRNA expression was elevated (29). In line with this, inhibition of MK, *via* an anti-N terminal domain of MK antibody, repressed neutrophil recruitment and NETosis in EAM mice (29). As a result of MK blockade, a reduction of fibrosis and preservation of systolic function was observed (29). Moreover, the N-terminal domain of MK is critical for MK-mediated leukocyte adhesion, because targeting it inhibited leukocyte adhesion as demonstrated through intravital microscopy of TNFα-treated cremaster muscle post-capillary venules (29). In extrapolation to our previous investigation (41), the MK-LRP1 axis in neutrophil recruitment was further examined. Using the aforementioned cremaster model in mice with a specific hematopoietic knock-out of *LRP1*, severely diminished leukocyte adhesion and neutrophil extravasation were revealed (29). Consistently, blocking LRP1 by its inhibitor receptor-associated protein also decreased leukocyte infiltration in EAM mice (29). *In vitro*, LRP1 and CD11a of the β_2_ integrin lymphocyte function–associated antigen 1 (LFA-1; CD11a/CD18), vital for neutrophil recruitment, specifically clustered and colocalized upon neutrophil adhesion (29). Finally, MK facilitated neutrophil trafficking and NET formation through LRP1 (29). In summary, the MK-LRP1 axis was essential for neutrophil recruitment and NET formation in myocardial inflammation and inhibiting MK might aid in treatment of myocarditis.

### Heart failure

Some studies focused on MK's participation in heart failure. In heart failure patients, serum MK concentrations were significantly higher, when compared to control subjects (117). The subgroup of patients who suffered cardiac events displayed significantly elevated MK concentration, as opposed to those who did not (117). This study suggests that MK levels were associated with cardiac events, establishing MK as an independent cardiac event predictor (117). Moreover, heart transplant recipients exhibited significantly increased MK levels, in comparison to the control group (118). MK serum concentrations demonstrated a positive correlation with advancing NYHA class and were associated with kidney function, transferrin, N-terminal prohormone of brain natriuretic peptide (NT-proBNP), and prednisone dose in these patients (118). Moreover, elevated urinary MK levels post-cardiac surgery were found to predict post-operative severe fluid overload (119).

Evaluation of MK's influence in heart failure pathophysiology delivered divergent results. One report demonstrated MK's harmful implication in cardiac hypertrophy (142). Upon assessment of microRNAs' role in cardiac hypertrophy, it was unraveled that miR-326 blunted cardiac hypertrophy through targeting MK and subsequently inhibiting the JAK-STAT and MAPK signaling pathways (142). In addition, lung and kidney MK expression were increased by pressure overload consequent to transverse aortic constriction (TAC) in mice (120). When performed in mice with cardiac-specific MK overexpression, TAC provoked ERK1/2 and Akt phosphorylation accompanied by a rise in fetal gene expression (120). These mice developed severe cardiac hypertrophy and fibrosis relative to wildtype mice (120). In line with this, they also presented with cardiac dysfunction and eventually progressed to congestive heart failure (120). Consequently, exacerbated survival rates 4 weeks post-surgery were observed in mice with cardiac-specific MK overexpression as opposed to wildtype mice (120). Moreover, in a study of cardiac remodeling in subtotal nephrectomy-induced chronic kidney disease, MK-deficient mice had lessened ERK1/2, Akt and EGFR phosphorylation and alleviated cardiac hypertrophy (143). Contiguously, MK treatment of neonatal rat cardiomyocytes induced opposite effects and EGFR inhibition suppressed MK's influence *in vitro* and *in vivo* (143). MK hence drived cardiac remodeling in chronic kidney disease *via* EGFR signaling (143). Furthermore, a different report disclosed MK protein upregulation in the sera and cardiac tissue of pediatric patients with dilated cardiomyopathy (DCM) (121). Whereas, MK treatment of neonatal rat ventricular myocytes upregulated atrial natriuretic factor (ANF) and BNP expression, incubation with DCM serum, supplemented with anti-MK antibody, averted ANF and BNP upregulation in these cells (121). Here, MK participated in the activation of fetal gene program mediated by DCM serum (121). However, and in divergence to the above presented findings, MK had a protective influence on cardiac remodeling in tachycardia-induced congestive heart failure (144). Subcutaneous MK application in rabbits with tachycardia-induced congestive heart failure led to heart function preservation and lesser mortality rate (144). In this regard, a drop in collagen deposition area, apoptotic cells and myocyte loss was noted (144). This cardioprotection was attributed to MK's anti-apoptotic function mediated by PI3K-Akt signaling (144). Although studied in zebrafish heart regeneration, which has limited direct implications in the human heart, Midkine-a expression was induced upon cardiac cryoinjury and was found to regulate the resultant fibrotic scar formation (122). In Midkine-a-deficient zebrafish, tissue regeneration was hindered *via* diminished EC proliferation and increased collagen deposition at the site of injury (122). In addition, a recent report exposed a novel role of the MK receptor PTPRZ1 in murine and zebrafish heart morphogenesis. In mice, PTPRZ1-deficiency resulted in left ventricular dilation, a decrease in ejection fraction and fraction shortening as well as induction of angiogenesis without cardiac hypertrophy (145). Also, PTPRZ1-deficient zebrafish presented with bradycardia, ventricular enlargement, defected contractility, and developmental cardiac markers' dysregulation (145). Therefore, PTPRZ1 seems to partake in cardiac morphogenesis and could play a role in idiopathic congenital cardiac pathologies (145). Nevertheless, it remains to be elucidated whether these PTPRZ1 functions are linked to MK and/or other PTPRZ1 ligands. In summary, MK became upregulated during heart failure and seems to have a detrimental function in cardiac hypertrophy, heart failure related to TAC-induced pressure overload and chronic kidney disease as well as pediatric DMC, while it had a preserving, protective effect in tachycardia-associated heart failure, contributed to zebrafish heart regeneration and might be involved in PTPRZ1-dependent cardiac morphogenesis.

### Sepsis

Sepsis is defined as a condition triggered by a dysregulation of host response to an infection leading to life-threatening organ dysfunction (146). Some studies explored MK expression in sepsis. Although with unclear total significance, high levels of serum MK were exhibited in patients with sepsis and septic shock (123). Further analysis showed that elevated MK levels were persistently measured in patients with cardiovascular insufficiency and mechanical ventilation, in contrast to decreasing levels in patients without these complications (123). In this study, significantly pronounced MK elevation was associated with gram-positive bacterial infections (123). As demonstrated by another report, significantly higher MK levels were measured in septic patients with moderate/severe acute respiratory distress syndrome (ARDS) as well as septic patients with acute kidney injury in comparison with patients without these complications, respectively (124). Moreover, MK plasma concentration was significantly elevated in the non-survivor group of septic patients, as opposed to the survivor group (124). MK levels thus correlated with pulmonary and renal injury as well as with 28-day mortality in patients with sepsis (124). Consistent with these findings, a recent report also delivered evidence of markedly raised MK levels in septic patients relative to healthy volunteers (48). In an attempt to elucidate MK's functional role in sepsis, the murine cecal ligation and puncture model of sepsis was put to use (48). Here, MK expression in plasma and in lung tissue was significantly augmented (48). Targeting MK with an adeno-associated virus, regionally in the lungs, alleviated lung injury in this model (48). Under these settings, MK instigated acute lung injury *via* activating the ACE system (48). Moreover, MK promoted vascular endothelial injury through induction of ACE expression *via* Notch2 (48). Therefore, this study illustrated a rather injurious role of MK in sepsis (48). On the other hand, given MK's role in host defense, as detailed earlier, one could assume that the upsurge in MK levels during sepsis could have anti-microbial benefits. However, one study found that MK's bactericidal effects declined when present in plasma (82). It has been proposed that MK's contribution to host defense takes place outside of the blood stream, which is supported by its constitutive expression at the body external barriers like large airways and skin (81). This suggests that the MK increase during sepsis is most probably part of a systematic response (81). Yet, it remains elusive whether MK plays an overall beneficial or harmful role in sepsis and additional investigations are required to clarify its impact in sepsis pathogenesis. Nevertheless, MK is definitely an attractive candidate for a potential new sepsis biomarker.

### Coronavirus disease 19

The rise of the coronavirus disease 19 (COVID-19) pandemic prompted many scientists and clinicians to speculate about MK's involvement in COVID-19 (147, 148). Infection with SARS-CoV-2 is the underlying cause of COVID-19 (149). The host reply during COVID-19 engages unhinged immune-inflammatory, thrombotic, and parenchymal responses (149). Some inquiries assessed MK expression in COVID-19. A recent report expounded upregulation of MK protein expression in plasma of COVID-19 patients (125). Also, another investigation described MK protein upregulation in sera of pregnant women with COVID-19, which was associated with pregnancy complications (126). In a similar study, MK serum levels in pregnant women with COVID-19 displayed a significant increase in contrast to healthy pregnant women (127). This augmented MK expression was significant throughout all trimesters (127). The mild group had profoundly lower MK levels when compared to the moderate or severe group (127). Also, MK levels positively correlated with disease severity and hospitalization length and negatively with the oxygen saturation (127). Hence, MK was upregulated in COVID-19 patients.

Some colleagues proposed few mechanisms through which MK might contribute to COVID-19 pathology. Probably the most momentous, if proven true, is MK's alleged facilitation of viral entry into target cells. The spike (S) protein present in SARS-CoV-2 enables its entry into target cells *via* interacting with ACE2, which converts angiotensin I and II to angiotensin-(1–9) and angiotensin-(1–7) respectively and is present on the surface of epithelial cells (150). This entry process is supported by transmembrane protease serine 2 (TMPRSS2) upon cell surface entry and cathepsin L for endosomal entry (150). It has been postulated that a MK-based complex with syndecan-1, glycosaminoglycans and heparan sulfate could enhance sulfatation sites of the receptor binding domain on the S protein (147). This in turn could promote ACE2 binding affinity (147). MK could thereafter mediate viral entry through LRP1-facilitated endocytosis (147). Taking into consideration the above-mentioned roles of MK in RAAS regulation and sepsis-associated lung injury as well as its implication in ARDS-related (47) and -unrelated lung fibrosis (99), it becomes quite plausible that MK might indeed play a role in COVID-19.

Perceived form a different angle, MK might contribute to COVID-19 pathology *via* its renowned inflammatory functions. A theory of MK stimulation of the presumed cytokine storm in COVID-19 (149) has been postulated (148). Here, the authors proposed that MK might contribute to the so-called cytokine storm characterized by excessive cytokine release. However, this proposal should be dealt with caution since according to the current scientific consensus, acceptance or rejection of the presumed cytokine storm in COVID-19 is yet to be decided (149). On another note, MK-mediated leukocyte recruitment, particularly of neutrophils, and NETosis might present another mechanism through which MK might aggravate COVID-19. Compellingly, several reports collectively delivered confirmation neutrophil involvement in COVID-19. Pulmonary neutrophil infiltration was illustrated in an autopsy specimen of a COVID-19 patient (151). Analysis of bronchoalveolar lavage fluid of COVID-19 patients divulged elevated chemokine levels, responsible for neutrophil recruitment (152). Notably, chemokine concentration even correlated with the viral load of SARS-CoV-2 (152). Moreover, markers of NET formation in the sera of COVID-19 patients were elevated (153). Also, COVID-19 patients' sera were capable of NETosis induction in control neutrophils (153). Further, activated neutrophils that featured a low-density phenotype, likely to undergo NETosis, were found in COVID-19 patients and elevated NET turnover markers were linked to disease severity (154). In microvessels originating from lungs and other organs of COVID-19 patients, NET aggregates were identified and were associated with endothelial damage and organ dysfunction (154). Hence, it is possible that MK-mediated neutrophil trafficking and NETosis might exacerbate lung damage, immunothrombosis and myocarditis in COVID-19.

## Conclusion

This review outlines the most significant, current findings on the roles of MK in cardiovascular diseases. MK not only became upregulated in cardiovascular diseases, it was implicated in their pathophysiology and possessed detrimental und beneficial functions alike. In summary, the evidence presented in this review lays the groundwork for establishing MK as a prospective and propitious diagnostic marker and therapeutic target in cardiovascular diseases.

## Author contributions

MM performed the literature search, data analysis and drafted the article. MM and LTW critically revised the work. Both authors read and approved the final manuscript.

## Funding

This work was supported by Sonderforschungsbereich 914 of the German Research Fund (Project B10 to LTW).

## Conflict of interest

The authors declare that the research was conducted in the absence of any commercial or financial relationships that could be construed as a potential conflict of interest.

## Publisher's note

All claims expressed in this article are solely those of the authors and do not necessarily represent those of their affiliated organizations, or those of the publisher, the editors and the reviewers. Any product that may be evaluated in this article, or claim that may be made by its manufacturer, is not guaranteed or endorsed by the publisher.
